# Outcomes of Surgical and Non-Surgical Treatment for Sixth Nerve Palsy

**Published:** 2010-01

**Authors:** Abbas Bagheri, Babak Babsharif, Mohammad Abrishami, Hossein Salour, Maryam Aletaha

**Affiliations:** Labbafinejad Medical Center, Shahid Beheshti University of Medical Sciences, Tehran, Iran

**Keywords:** Abducens Nerve Diseases, Strabismus

## Abstract

**Purpose:**

To report the outcomes of surgical and non-surgical treatment in sixth nerve paresis and palsy.

**Methods:**

This retrospective study was performed on hospital records of 33 consecutive patients (37 eyes) with sixth nerve dysfunction who were referred to Labbafinejad Medical Center from September 1996 to September 2006, and underwent surgical procedures or botulinum toxin injection. Patients were divided into three groups: group A had muscle surgery without transposition, group B underwent transposition procedures and group C received Botulinum toxin injection.

**Results:**

Overall, 33 patients including 19 male and 14 female subjects with mean age of 20.4±17.2 years (range, 6 months to 66 years) were studied. Eye deviation improved from 50.3±16.8 to 6.0±9.8 prism diopters (PD) after the first operation and to 2.5±5.0 PD after the second operation in group A, from 56.9±24.3 to 5.5±16.0 PD after the first procedure and to almost zero following the second in group B, and from 44.3±10.5 to 15.0±20.0 PD 6 months following botulinum toxin injection in group C. Head posture and limitation of motility also improved significantly in all three groups. The overall rate of reoperations was 21%.

**Conclusions:**

Various procedures are effective for treatment of sixth nerve dysfunction; all improve ocular deviation, head turn and abduction deficit. The rate of reoperation is not high when treatment is appropriately selected according to clinical condition.

## INTRODUCTION

Paralytic strabismus poses a significant diagnostic and therapeutic challenge. Congenital and acquired sixth nerve palsy has been shown to be the most common type of cerebral nerve palsy in some studies,^1^ comprising up to 45 percent of referrals for cerebral nerve palsy.^2^ Due to its long intracranial path, its angle at the petrous tip of the temporal bone and non-flexibility of the path between the brainstem and the meningeal entrance site; the abducens nerve is susceptible to damage with various types of intracranial pathologies such as meningeal inflammation or edema and any displacement of the brain stem. The sixth cranial nerve, similar to other cranial nerves, is also sensitive to toxic substances, demyelinative processes and viral diseases.^3^

Congenital sixth nerve paresis is uncommon and difficult to distinguish from congenital esotropia, Duane’s syndrome and Mobius syndrome.^4^ The acquired variant improves spontaneously over 3 to 6 months; based on current information, 78% of patients recover in one year and 40% of the remaining cases have a severe underlying condition such as intracranial aneurysms or vascular disorders (brainstem stroke), carotid-cavernous fistula or cerebral tumors.^5^–^6^

In patients with slow improvement, contracture of the medial rectus muscle may result in concomitant esotropia with positive forced duction test (FDT). After identifying the underlying cause, several interventions such as patching, compensatory prisms or Botulinium toxin injections are used to prevent diplopia and abnormal head posture in the first 6 months following sixth nerve palsy. When esotropia is not improving and becomes stable for at least 6 months, surgical intervention is warranted. A variety of procedures may be performed such as recession and resection (R&R), bilateral medial rectus recession (BMR), different muscle transposition procedures (Hummelschiem and Jensen) with or without medial rectus (MR) recession, and bilateral lateral rectus (LR) resection along with medial rectus myotomy.

In this study, we present the clinical features of a consecutive series of patients with sixth nerve palsy over a 10-year period at Labbafinejad Medical Center (LMC) and the outcomes of medical and surgical treatment for this condition.

## METHODS

This retrospective study was performed on hospital records of consecutive patients with sixth nerve palsy who were referred to LMC, Tehran, Iran from September 1996 to September 2006. Relevant data included age, gender, reason for referral, laterality of the palsy, angle of deviation, head posture, limitation of motility (from −1 to −4; indicating abduction deficit of 25% to 100%), positive FDT, type of treatment (surgical versus non-surgical), and etiology of sixth nerve dysfunction (congenital, traumatic, ischemic, infectious, tumoral or inflammatory). The type and number of operations were also determined which included R&R, BMR, MR myotomy, LR resection, full tendon transposition with or without MR recess and Jensen’s procedure. Patients were followed one day, one and 6 weeks, and 3 months after surgery. Further follow-up visits were arranged based on the condition of the individual patient.

Visual acuity was measured using Snellen optotypes where possible; fixation patterns were used for preverbal children to determine centration, steadiness and maintenance (CSM) of fixation.

Patients were divided into 3 different groups ; group A underwent surgical intervention without transposition, group B underwent muscle transposition, and group C received one dose (10–20 unit) of Botulinum toxin (Dysport, Ipsen, Slough, UK) injected into the MR muscle. Data were compared pre-and postoperatively using paired *t-*test with significance level set at P<0.05.

## RESULTS

**Figure 1:**
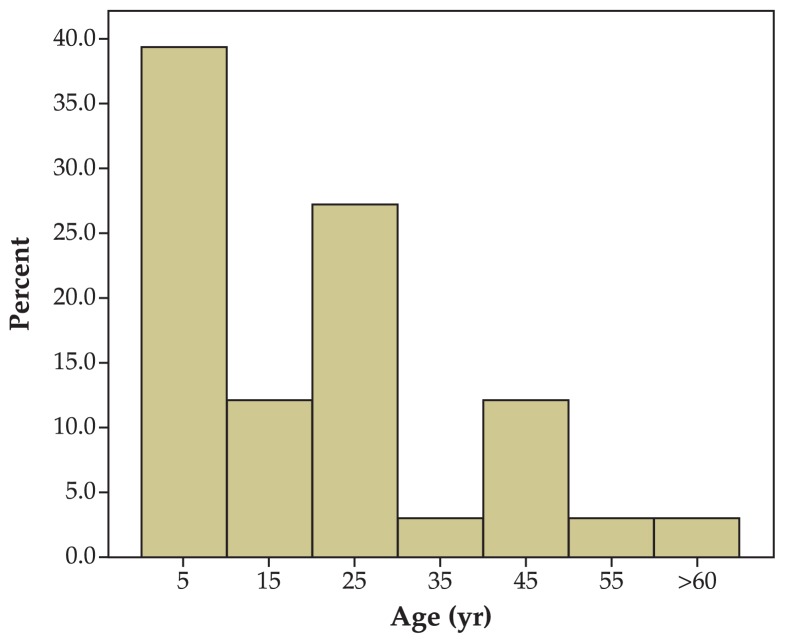
Patients’ age distribution.

**Figure 2:**
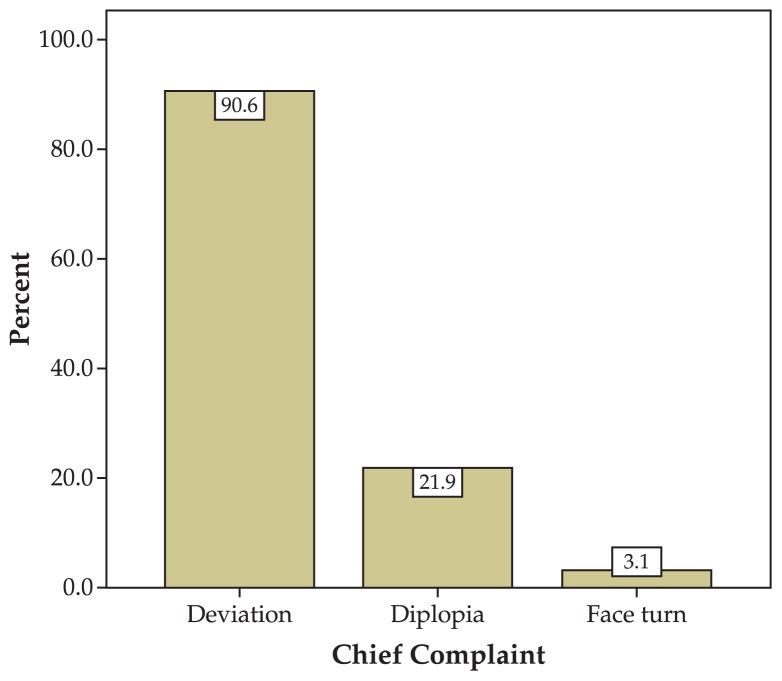
Chief complaints of the patients.

**Figure 3:**
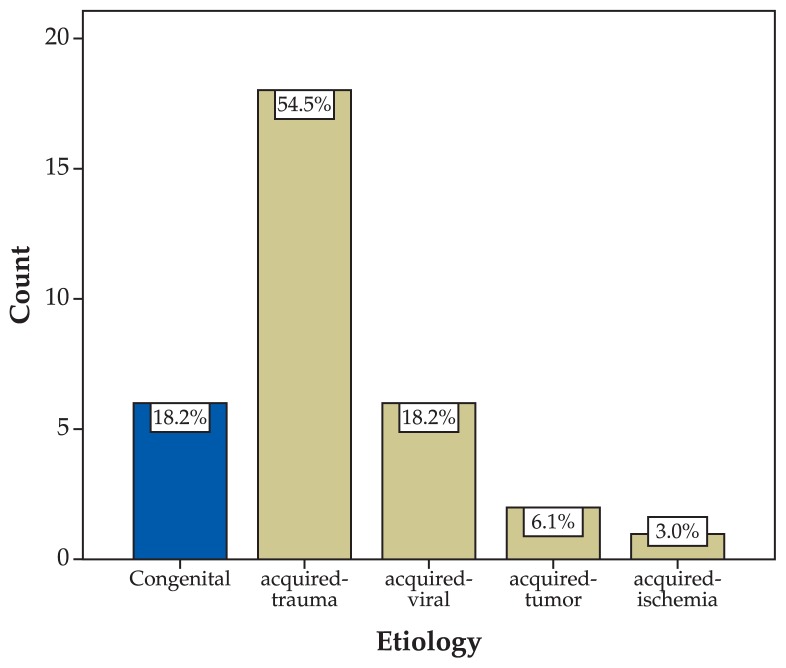
Etiology of sixth nerve palsy.

This study included 33 patients consisting of 19 (57.6%) male and 14 (42.4%) female subjects with mean age of 20.4±17.2 years (range, 6 months to 66 years) (Fig. 1). Mean follow-up was 17.1±25.3 months and for groups A, B, and C, 19.3±31.9, 18.1±21.6, and 11.2±10.2 months respectively. The right eye was involved in 10 (30.3%) subjects, the left eye in 19 (57.6%) patients and both eyes in the other 4 (12.1%) individuals. Chief complaints included squint in 30 (91.0%) cases, diplopia in 7 (21.2%) cases and abnormal head posture in one (3.0%) patient (Fig. 2). The sixth nerve dysfunction was traumatic in 18 (54.4%) cases, congenital in 6 (18.2%) patients, due to viral infections in 6 (18.2%) individuals, secondary to tumors in 2 (6.1%) subjects, and ischemic in 1 (3%) patient (Fig. 3).

Regarding treatment, 17 cases (51.5%) were operated without muscle transposition (group A), 8 subjects (24.2%) underwent muscle transposition (group B), and 8 individuals (24.2%) received Botulinum toxin injections (group C). Seven (21%) patients required a second procedure including 4 cases in the transposition group, 2 in the R&R group and one in the Botulinium group. Table 1 shows mean eye deviation before and after the first and second operations. Preoperatively, mean deviation was 50.3±17.7 (range, 18–100) PD overall, which was reduced to 9.0±13.8 PD (range, 50 PD esotropia to 16 PD exotropia) after the first operation, and 1.7±3.1 (range, 0–10) PD after the second procedure (Table 1, P<0.001). In the transposition group, 4 patients received full tendon transposition with MR recess, 3 patients underwent full tendon transposition without MR recess, and one patient underwent Jensen’s procedure together with MR recess.

**Figure 4:**
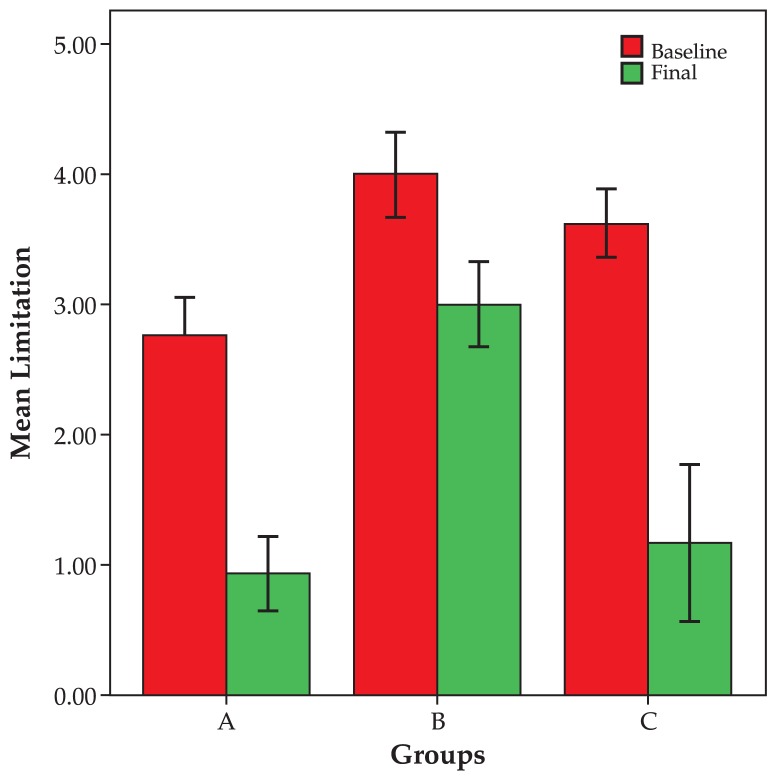
Changes in limitation of eye movements in different study groups.

Mean preoperative amount of head posture was 15.0±8.9, 11.3±6.3 and 15.0±7.1 degrees in groups A, B and C respectively which reached zero postoperatively (P= 0.03, 0.07 and 0.20 respectively). Preoperatively, mean limitation of motility was −2.7±1.2 in the non-transposition group, −4.0±0.9 in the transposition group and −3.6±0.7 in the Botulinium group, which reached −9.0±1.0 (P=0.002), −3.0±0.9 (P=0.02) and −1.2±1.4 (P=0.04), respectively (Fig. 4).

**Figure 5:**
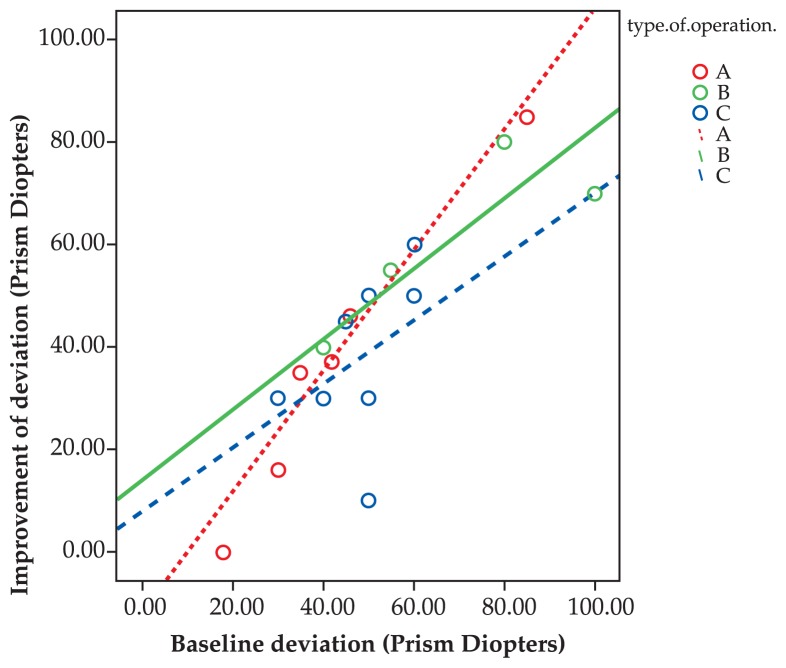
Correlation between baseline eye deviation and postoperative improvement. Group A (R^2^: 0.945, P<0.001) Group B (R^2^: 0.836, P: 0.001) Group C (R^2^: 0.177, P: 0.299)

**Figure 6:**
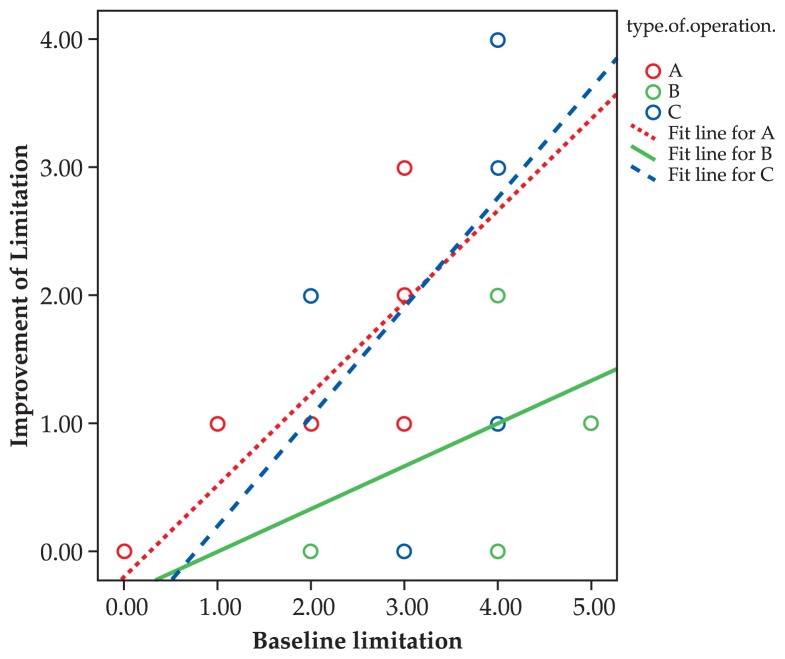
Correlation between baseline limitation and postoperative improvement. Group A (R^2^: 0.395, P: 0.012) Group B (R^2^: 0.167, P: 0.315) Group C (R^2^: 0.193, P: 0.384)

**Figure 7:**
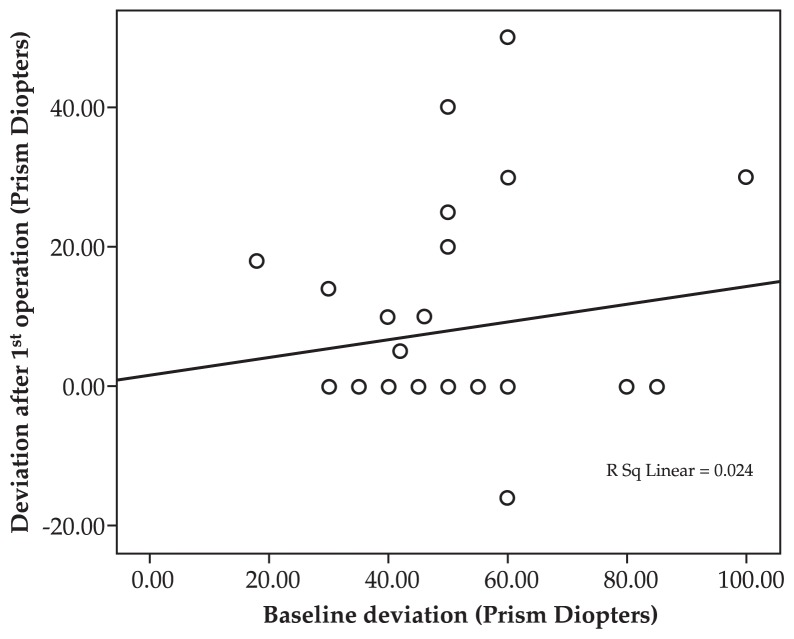
Correlation between primary and residual deviation after first surgery.

Comparing the outcomes of treatment among the three groups, improvements in eye deviation (P=0.3) and head posture (P=0.9) were not significantly different. However, ocular deviation was significantly less corrected with muscle transposition (P=0.001). The greater the primary deviation and limitation of motility, the larger the improvement (Figures 5 and 6, Spearman correlation). Furthermore, the higher the primary deviation, the better the response to primary strabismus surgery (Fig. 7, Spearman correlation).

## DISCUSSION

Sixth nerve palsy has been reported to be the most common type of extraocular nerve paralysis based on some studies^2^,^7^, but ranking second following fourth nerve palsy according to other reports.^8^–^10^ This condition has been reported to be more prevalent in male subjects^7^,^10^ which may be due to the higher rate of trauma in men, especially accidents and work related injuries.

Among acquired cases of sixth nerve palsy in our series, trauma was the most common cause followed by viral infections, tumors and cerebral ischemia. Although more than 80% of patients had acquired dysfunction, the most prevalent chief complaint was squint rather than diplopia. This may be explained by the young patient population (most subjects were in their first decade of life) with higher potential for suppression. We have shown in two previous reports from our center that congenital causes of palsies comprise less than 20% of all cases.^7^,^10^ Ariochane^11^ studied 64 patients with sixth nerve dysfunction less than 7 years of age and reported hydrocephalus as the most common cause followed by trauma, congenital conditions and viral infections. In a study on 132 patients younger than 18 years with sixth nerve palsy, Afifi et al^12^ reported the causes in descending order: trauma, tumors, congenital disorders, viral infections and hydrocephalus. In a study by Robertson et al^13^ on 103 patients under 15 years of age, the leading cause was tumors followed by trauma, inflammation and idiopathic. Mittleman et al,^14^ in a study on 64 children under 7 years, observed the most common causes to be tumors, hydrocephalus and trauma.

The highest success rate was gained after primary intervention such that only 21% of our patients required a second intervention, most in the transposition group (4 cases). After the first operation, the best result was observed in the group with no transposition and the worst response was found in the Botulinum group. Therefore, it may be concluded that proper selection of treatment based on the severity of paresis, its onset and the amount of motility limitation can increase the chance for an optimal response. The most significant level of limitation of motility was observed in the transposition group. As expected, the most significant level of improvement in limitation was gained with non-transposition procedures, while the Botulinium group had an intermediate situation.

As shown in figure 2, the more severe the primary deviation, the greater the improvement after surgery. In other words, strabismus surgery has a self-adjustable nature, i.e. the same procedure results in greater response with more severe strabismus. However, as the primary deviation increases, residual deviation also increases. When primary limitation of motility increases, improvement also increases after surgery; a steeper slope was observed in the non-transposition group. Despite improvement in primary position deviation, eyes in the transposition group which were completely paralyzed had the least amount of improvement in limitation of motility.

The significant improvement in the Botulinium group reflects the possibility of utilizing minimally invasive methods such as Botulinum toxin. In a study on 33 patients with a clinical diagnosis of acute traumatic sixth nerve paresis, Holmes et al^15^ showed that 86% of unilateral and 38% of bilateral cases improved spontaneously after 3 months and recommended that Botulinum toxin can prevent MR fibrosis during the period of self-improvement.

In conclusion, the effect of strabismus surgery in sixth nerve palsy appears to be self-adjustable and if the procedure is selected appropriately, the success rate is high with only one patient out of five requiring a reoperation.

## Figures and Tables

**Table 1:** Preoperative and postoperative mean ± SD ocular deviation (prism diopters) in different groups

Groups	Preoperation	After first operation	After second operation
A (Non-transposition)	56.9±24.3	5.5±16.0	0
B (Transposition)	50.3±16.8	6.0±9.8	2.5±5.0
C (Botulinium*)	44.3±10.5	15.0±20.0	0
Total	50.3±17.7	9.0±13.8	1.7±3.1

SD, standard deviation;

* only one patient in this group required a second injection.
